# Prognostic Factors of Survival with Aflibercept and FOLFIRI (fluorouracil, leucovorin, irinotecan) as Second-line Therapy for Patients with Metastatic Colorectal Cancer

**DOI:** 10.7150/jca.49176

**Published:** 2021-01-01

**Authors:** Jinchul Kim, Hana Kim, Jung Yong Hong, Jeeyun Lee, Se Hoon Park, Joon Oh Park, Young Suk Park, Ho Yeong Lim, Won Ki Kang, Seung Tae Kim

**Affiliations:** 1 Division of Hematology-Oncology, Department of Medicine, Samsung Medical Center, Sungkyunkwan University School of Medicine, Seoul, Korea.; 2 Department of Hematology-Oncology, Inha University College of Medicine and Hospital, Incheon, Korea.

**Keywords:** Aflibercept, colorectal cancer, prognostic factor

## Abstract

**Background:** Aflibercept and fluorouracil, leucovorin, irinotecan (FOLFIRI) is commonly used as a second-line treatment for metastatic colorectal cancer (CRC). However, the biomarkers to guide the choice of this regimen from among treatment options remain unclear.

**Patients and Methods:** We performed exploratory analyses to validate potential prognostic factors for patients receiving aflibercept plus FOLFIRI as a second-line systemic treatment for metastatic CRC between January 2015 and July 2019. Patient characteristics, histopathologic data, laboratory and radiologic data, and treatment outcomes were collected and reviewed.

**Results:** Included were 52 patients: 50 (96.2%) received bevacizumab plus fluorouracil, leucovorin, oxaliplatin (FOLFOX) as prior first-line treatment. Among the 52 patients receiving aflibercept and FOLFIRI, four complete responses and 21 partial responses were observed in analyzed patients for an overall response rate of 48.1%. Median progression-free survival (PFS) was 7.0 months and overall survival (OS) was 16.8 months. Response to first-line treatment (median PFS, 8.0 versus 4.2 months), left-side location of primary tumor (7.9 versus 4.9 months), low baseline CEA level (8.0 versus 5.9 months), and no RAS/RAF mutation (9.9 versus 6.4 months) were remained significant prognostic factors for PFS in the multivariate backward stepwise Cox regression model, and the latter three factors were also significantly related to OS.

**Conclusions:** Significant prognostic factors for PFS with aflibercept plus FOLFIRI as second-line therapy were extracted and validated in the multivariate OS model. These findings could provide useful information for selecting patients for aflibercept plus FOLFIRI as second-line therapy.

## Introduction

Colorectal cancer (CRC) is the fourth leading cause of cancer-related deaths in men and the third leading cause of cancer-related deaths in women worldwide 1. Approximately 30% of CRC patients present with metastatic disease at the time of diagnosis, and 50% with localized tumors will develop metastasis later after surgery 2. Despite recent advances in treatment modalities, the 5-year survival rate for metastatic CRC is approximately 14% 3.

Standard palliative chemotherapy for metastatic CRC is a fluorouracil-based treatment combination with oxaliplatin or irinotecan, and with or without biological agents including anti-angiogenesis or anti-epidermal growth factor receptor (EGFR) monoclonal antibodies. The majority of patients are treated with a biological agent as first-line systemic treatment mainly according to RAS/RAF mutation status of tumors 4. Current guidelines recommend bevacizumab plus chemotherapy for KRAS/NRAS mutated tumors and either an anti-EGFR agent or bevacizumab for KRAS/NRAS wild-type tumors as first-line treatment options 5. Second-line treatment often depends on first-line chemotherapy regimens. Fluorouracil, leucovorin, irinotecan (FOLFIRI) with biological agents as second-line therapy can be used with patients with failed fluorouracil, leucovorin, oxaliplatin (FOLFOX) or capecitabine, oxaliplatin (CAPEOX) plus bevacizumab treatment, which are some of the most commonly used first-line combination treatments.

Aflibercept, a recombinant anti-angiogenic protein, selectively blocks VEGF-A, VEGF-B, and placental growth factor 6, precluding downstream effects including angiogenesis and metastasis. This effect is different from bevacizumab, which selectively inhibits only VEGF-A. The phase III VELOUR trial evaluated the efficacy of aflibercept plus FOLFIRI as second-line treatment for patients with metastatic CRC who had progression following an oxaliplatin-based regimen, with or without bevacizumab 7. The study found that aflibercept plus FOLFIRI significantly prolonged median overall survival (OS) (13.5 versus 12.1 months) and progression-free survival (PFS) (6.9 versus 4.7 months) compared to the control group.

Although there is a need for consideration of some issues like molecular profile, performance status, comorbidities, and sidedness of tumor, bevacizumab plus oxaliplatin-based regimen is thought to be the most commonly used combination as front-line systemic chemotherapy for metastatic CRC 8, 9. It has some advantages over anti-EGFR agents even in RAS/RAF wild type tumor; there is no waiting turnaround time for molecular analysis, and relatively lower toxicity was reported in several studies 10, 11. As aflibercept plus FOLFIRI is the practical alternative for bevacizumab plus FOLFIRI as second-line treatment in this setting, we analyzed the real-world treatment outcome of second-line aflibercept plus FOLFIRI for metastatic CRC patients, the majority of whom received bevacizumab plus FOLFOX previously. Additionally, exploratory analyses to investigate potential biomarkers to predict the clinical efficacy of the combination treatment were performed.

## Methods

### Patients and variables

Patients with metastatic CRC who received a regimen of aflibercept plus FOLFIRI as a second-line therapy at Samsung Medical Center (Seoul, Korea) and Inha University Hospital (Incheon, Korea) between January 2015 and July 2019 were included. Medical records of patients were retrospectively collected for age, gender, Eastern Cooperative Oncology Group (ECOG) performance status, outcomes after first-line treatment, previous surgery for CRC, location of primary tumors, metastasis sites, number of metastatic sites, baseline carcinoembryonic antigen (CEA) level (at time of second-line therapy initiation), RAS/BRAF mutation status, microsatellite instability status and treatment outcomes with aflibercept plus FOLFIRI.

Patients were eligible when they had been treated with second-line aflibercept plus FOLFIRI as part of routine clinical practice. Patients who had received prior irinotecan-containing regimens or aflibercept plus chemotherapy other than FOLFIRI were excluded.

Overall response was classified as complete response (CR), partial response (PR), stable disease (SD), or progressive disease (PD) according to the Response Evaluation Criteria in Solid Tumors guidelines version 1.1. To protect the personal information of patients and privacy of the data, all data were handled anonymously. The trial protocol was approved by the institutional review board of Samsung Medical Center and Inha University Hospital and conducted in accordance with the Declaration of Helsinki.

### Statistical analysis

Descriptive statistics were calculated as a form of proportions and medians. Fisher's exact test was used to compare proportions, and Wilcoxon rank-sum test was used to compare continuous variables. PFS was defined as the interval from the start of treatment to the date of disease progression or death of any cause, and OS as the interval from the start of treatment to the date of death due to any cause. Kaplan-Meier curves and *p*-values calculated from log-rank tests were used to compare PFS between pre-defined subgroups. Both univariate and multivariate analyses using the Cox proportional hazards regression model were applied to estimate each factor's hazard ratio and corresponding confidence intervals. Two-sided *p*-values of 0.05 or lower were considered to indicate statistical significance. R studio software, version 1.2.1335, was used to perform all statistical analyses.

## Results

### Clinicopathological characteristics of patients

A total of 52 patients, 46 from Samsung Medical Center and 6 from Inha University Hospital, with metastatic CRC receiving aflibercept plus FOLFIRI as second-line therapy were analyzed. Patient baseline characteristics according to response to aflibercept plus FOLFIRI are in Table 1. The median age was 59.0 years (interquartile range, 51-67 years) with 24 (46.2%) males and 28 (53.8%) females. As prior first-line treatment, two (3.8%) patients received CAPEOX and all other patients received bevacizumab plus FOLFOX. Thirty-two (61.5%) patients had surgery for CRC and metastatic sites if possible, and 30 (57.7%) patients had tumors with mutations in the RAS/RAF pathway, while RAS/RAF mutational status was not available for three (5.8%) patients.

### Treatment Outcomes

**Figure 1:**
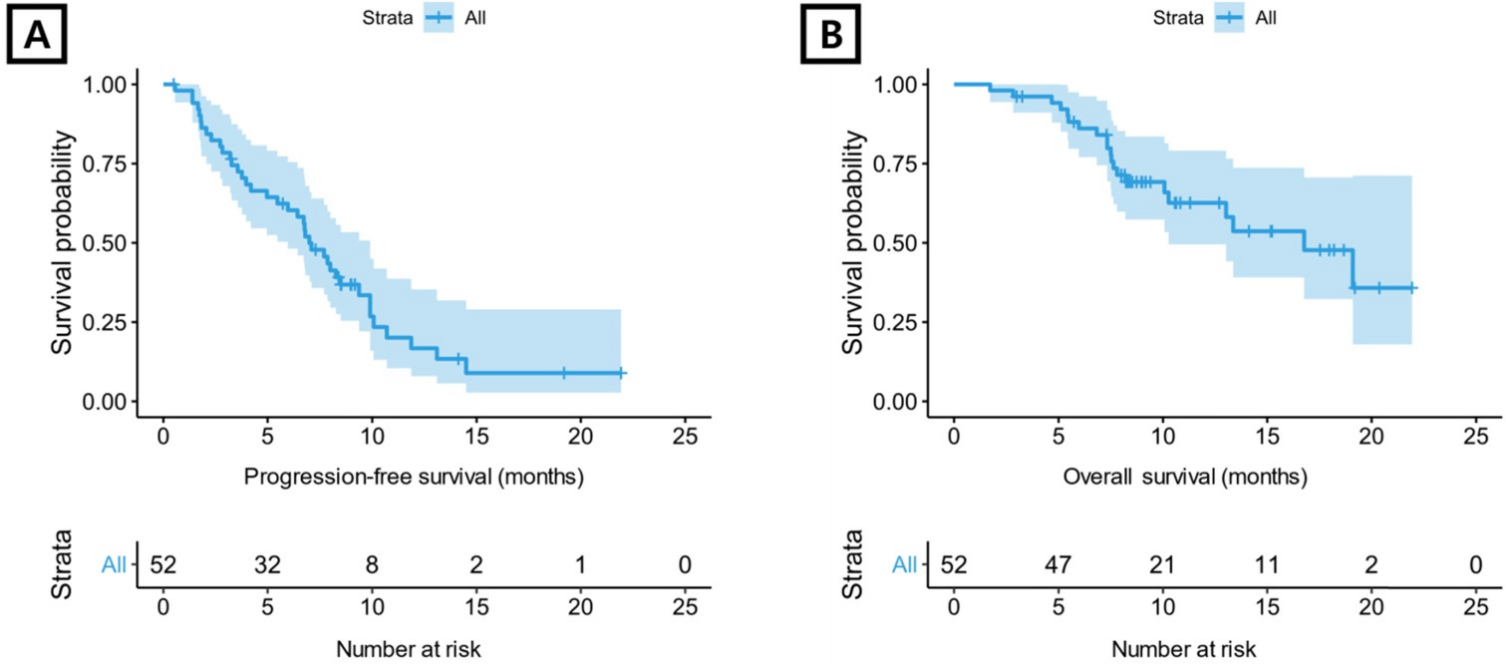
Kaplan-Meier plot. (A) progression-free survival and (B) overall survival for total included patients.

All 52 patients were evaluable for tumor response (Supplementary Table S1). Four (7.7%) patients demonstrated a CR, and a PR was reported in 21 (40.4%) patients. The overall response rate was 48.1%, and the disease control rate was 84.6%. For aflibercept plus FOLFIRI, median PFS was 7.0 months (95% confidence interval [CI] 6.0-9.9 months) (Figure 1A) and median OS was 16.8 months (95% CI 10.3-not reached) (Figure 1B).

**Figure 2:**
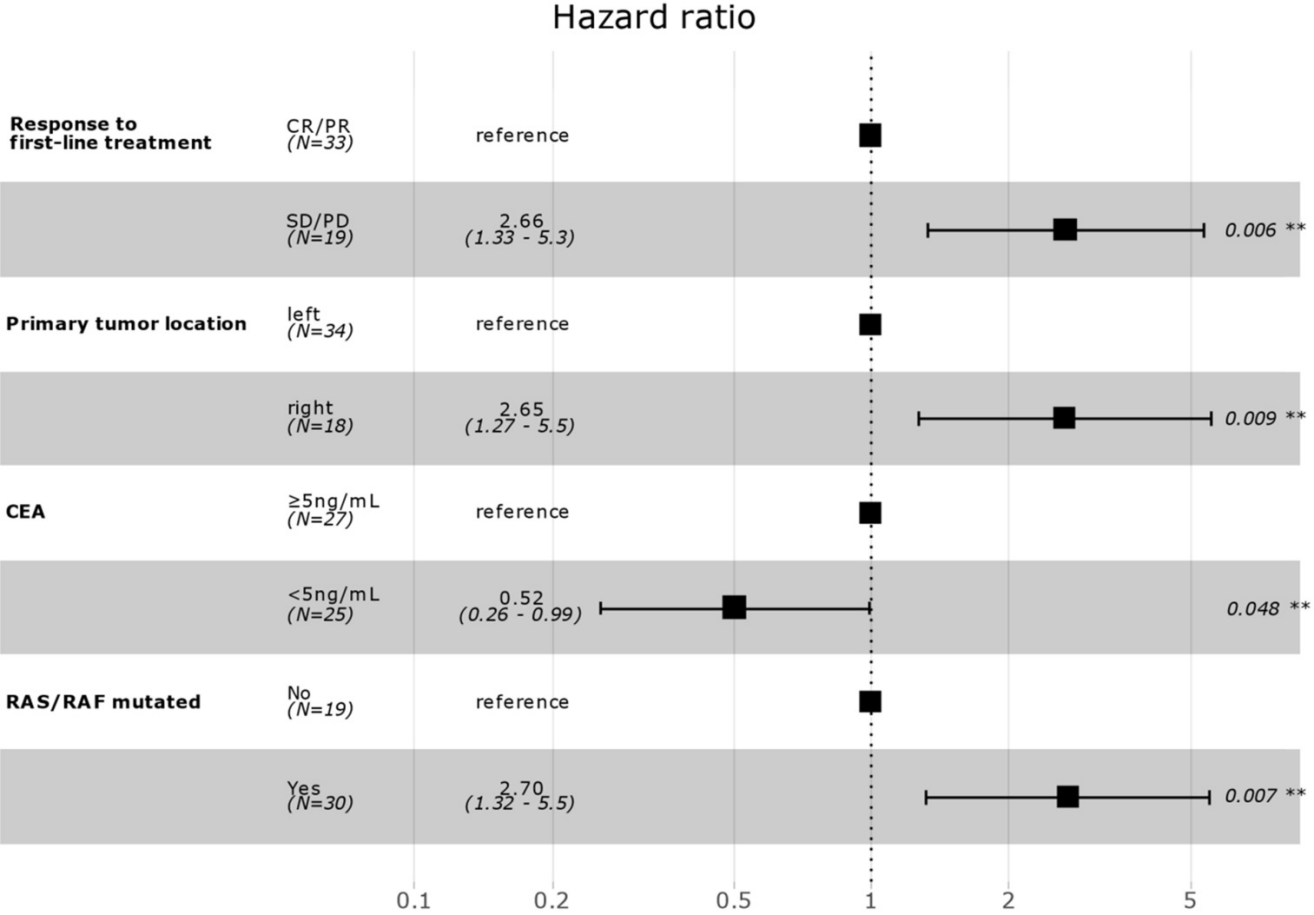
Multivariate survival model after variable selection. CR, complete response; PR, partial response; SD, stable disease; PD, progressive disease; CEA, carcinoembryonic antigen.

Correlation between patient clinicopathological characteristics and PFS was assessed to investigate potential biomarkers that could predict the effects of aflibercept plus FOLFIRI. In univariate Cox regression analysis, response to first-line treatment (non-responder, SD/PD versus responder, CR/PR), previous surgery for disease (yes versus no), primary tumor location (right versus left), number of organ metastasis (≥2 versus 1), baseline CEA level (<5 ng/mL versus ≥5 ng/mL), and RAS/RAF mutation (yes versus no) were significantly associated with PFS (Table 2). In multivariate backward stepwise Cox regression analysis including variables with *p*-value < 0.1 on univariate analysis showed that response to first-line therapy (median PFS, 8.0 versus 4.2 months), left-side location of primary tumor (7.9 versus 4.9 months), low baseline CEA level (8.0 versus 5.9 months), and no RAS/RAF mutation (9.9 versus 6.4 months) were independently correlated with PFS in the final model (Figure 2). Kaplan-Meier survival curves for PFS for the variables are in Supplementary Figure S1-3.

In the univariate Cox regression model for OS, previous surgery for the disease (yes versus no) and baseline CEA level were significantly correlated with OS. The multivariate backward stepwise Cox regression model including variables with P<0.1 on univariate analysis demonstrated that location of primary tumor, baseline CEA level, and RAS/RAF mutational status remained in the final model (Supplementary Figure S4).

## Discussion

This study provides clinical-pathological biomarkers that are easily available in clinical practice to select a CRC patient population that is likely to benefit from aflibercept plus FOLFIRI as second-line therapy. Response to first-line treatment (median PFS, 8.0 versus 4.2 months), left-side location of primary tumor (left, 7.9 versus right, 4.9 months), low baseline CEA level (8.0 versus 5.9 months), and no RAS/RAF mutation (9.9 versus 6.4 months) were significant prognostic factors for PFS to aflibercept plus FOLFIRI. Furthermore, the location of the primary tumor, baseline CEA level, and RAS/RAF mutational status were also significantly related to OS. This finding might be helpful for deciding among options for second-line treatment.

CEA is highly upregulated by various cancers and in about three-fourths of metastatic CRC patients 12. Baseline increased CEA levels correlate with poor prognosis 13. In a previous study on the predictive role of baseline CEA level for FOLFIRI plus cetuximab or bevacizumab as first-line therapy, high CEA level was associated with shorter OS in patients receiving FOLFIRI plus bevacizumab. The finding was not inconsistent with findings for patients receiving FOLFIFI plus cetuximab 14. Fiala et al. reported that baseline CEA level does not affect PFS or OS of patients treated with chemotherapy plus an anti-EGFR agent in combination 15. Another study on a proposed prognostic nomogram for FOLFIRI plus aflibercept included baseline CEA level as a significant influencer 16. These observations might be explained by that CEA exerts angiogenesis-promoting effects independent of VEGF, and increased CEA levels could achieve some angiogenic properties even when the VEGF pathway is blocked 17, 18. In our study, low baseline CEA level (<5 ng/mL) was significantly associated with prolonged median PFS with FOLFIRI plus aflibercept compared to high CEA level (≥5 ng/mL) (8.0 versus 5.9 months), consistent with the other studies.

Mutations in RAS and/or BRAF genes are associated with a lack of treatment response to anti-EGFR agents, and those mutations are also known to be related to inferior survival of patients with metastatic CRC 19. According to a previous study on the impact of KRAS, NRAS and BRAF mutations in tumors of patients with metastatic CRC receiving first-line therapy, the negative prognostic value of KRAS/BRAF mutations was observed in both bevacizumab-treated and non-bevacizumab-treated groups. However, the prognostic impact was more substantial in the bevacizumab-treated group than the non-bevacizumab-treated group. The hazard ratios for KRAS mutation versus wild type were 1.33 and 1.05, and BRAF mutation versus wild type were 2.61 versus 1.55 in the bevacizumab-treated group and the non-bevacizumab-treated group, respectively 19. Similar trends were observed in the biomarkers sub-analyses of the Velour trial 20 and a study of a prognostic nomogram for FOLFIRI plus aflibercept 16. Since mutations in RAS and BRAF are usually mutually exclusive 21 and have the same negative predictive value for anti-EGFR antibody use, several studies used these genes as one category of predictive variables, as we did. In our study, having no RAS/RAF mutation was significantly related to prolonged median PFS compared to presence of the RAS/RAF mutation (9.9 versus 6.4 months), so this could be a prognostic biomarker for use of FOLFIRI plus aflibercept.

The location of the primary tumor is an important prognostic factor in CRC due to distinct biological features of right-sided and left-sided tumors 22. Right-sided colon cancer is associated with defective mismatch repair genes and KRAS/BRAF mutations. Left-sided CRC is related to chromosomal instability and mutations in p53 and NRAS 23. A previous study to investigate the impact of primary tumor location on the efficacy of adding bevacizumab to chemotherapy for metastatic CRC reported that PFS improved with bevacizumab plus chemotherapy versus chemotherapy alone for right-sided tumors (hazard ratio = 0.75; p = 0.008) and left-sided (hazard ratio = 0.76; *p* < 0.001) tumors. The study concluded that the effect of bevacizumab was independent of tumor location 24. However, another study on the impact of primary tumor sidedness on treatment outcomes for patients receiving bevacizumab-based therapies found that the efficacy of bevacizumab in patients with a left-sided tumor is superior to efficacy for right-sided tumors, particularly in a wild-type RAS/BRAF subgroup 25. Comparable results were also reported in aflibercept plus FOLFIRI-treated groups 16, 26. In our study, left-sided primary tumor location was a significant factor for prolonged median PFS compared with right-sided tumor location (7.9 versus 4.9 months). Therefore, based on these results, primary tumor location is a prognostic factor for survival with aflibercept plus FOLFIRI.

This study has several limitations. First, a relatively small number of patients treated with aflibercept plus FOLFIRI as a second-line therapy were included. Additionally, due to a few missing observations for RAS/RAF mutation status, these mutation profiles were handled as the same group. Next, the retrospective characteristics of the study and exploratory analyses are subject to potential biases from a statistical perspective. Third, the number of factors included in the multivariate analyses was relatively high, considering the total number of patients and events. Despite these inherent limitations, our study analyzed and validated prognostic factors for survival outcomes with aflibercept plus FOLFIRI in the CRC patients who received bevacizumab plus FOLFOX as first-line therapy. These findings could provide useful information for selecting patients for aflibercept plus FOLFIRI as second-line therapy.

## Supplementary Material

Supplementary figures and tables.

## Figures and Tables

**Table 1:** Baseline characteristics

Overall response	Responders (N=25)	Non-responders (N=27)	Total (N=52)
Median age, years (IQR)	57.0 (50.0;66.0)	63.0 (54.0;67.0)	59.0 (51.0;67.0)
**Gender**			
Female	14 (56.0%)	14 (51.9%)	28 (53.8%)
Male	11 (44.0%)	13 (48.1%)	24 (46.2%)
**ECOG performance**			
0	9 (36.0%)	6 (22.2%)	15 (28.8%)
1	15 (60.0%)	21 (77.8%)	36 (69.2%)
2	1 (4.0%)	0 (0.0%)	1 (1.9%)
**First-line regimen**			
Bevacizumab + FOLFOX	24 (96.0%)	26 (96.3%)	50 (96.2%)
Capecitabine + oxaliplatin	1 (4.0%)	1 (3.7%)	2 (3.8%)
**Response to first-line treatment**			
CR/PR	19 (76.0%)	14 (51.9%)	33 (63.5%)
SD/PD	6 (24.0%)	13 (48.1%)	19 (36.5%)
**Previous anti-angiogenic treatment**			
No	1 (4.0%)	1 (3.7%)	2 (3.8%)
Yes	24 (96.0%)	26 (96.3%)	50 (96.2%)
**Previous surgery**			
No	5 (20.0%)	15 (55.6%)	20 (38.5%)
Yes	20 (80.0%)	12 (44.4%)	32 (61.5%)
**Primary tumor location**			
Ascending colon	6 (24.0%)	11 (40.7%)	17 (32.7%)
Descending colon	1 (4.0%)	0 (0.0%)	1 (1.9%)
Rectosigmoid colon	18 (72.0%)	16 (59.3%)	34 (65.4%)
**Metastasis to liver**			
No	8 (32.0%)	7 (25.9%)	15 (28.8%)
Yes	17 (68.0%)	20 (74.1%)	37 (71.2%)
**Metastasis to lung**			
No	17 (68.0%)	14 (51.9%)	31 (59.6%)
Yes	8 (32.0%)	13 (48.1%)	21 (40.4%)
**Metastasis to bone**			
No	22 (88.0%)	26 (96.3%)	48 (92.3%)
Yes	3 (12.0%)	1 (3.7%)	4 (7.7%)
Baseline median CEA level, ng/mL (IQR)	3.2 (1.5;5.9)	12.7 (5.3;129.7)	5.5 (2.5;40.5)
**Baseline CEA level**			
≥5 ng/mL	7 (28.0%)	20 (74.1%)	27 (51.9%)
<5 ng/mL	18 (72.0%)	7 (25.9%)	25 (48.1%)
**Mutation of RAS/RAF**			
No	16 (64.0%)	3 (11.1%)	19 (36.5%)
Yes	8 (32.0%)	22 (81.5%)	30 (57.7%)
Non-available	1 (4.0%)	2 (7.4%)	3 (5.8%)
**Peritoneal seeding**			
No	17 (68.0%)	16 (59.3%)	33 (63.5%)
Yes	8 (32.0%)	11 (40.7%)	19 (36.5%)
**MSI status**			
MSS	25 (100%)	27 (100%)	52 (100%)

CR, complete response; PR, partial response; SD, stable disease; PD, progressive disease; ECOG, Eastern Cooperative Oncology Group; FOLFOX, fluorouracil plus leucovorin plus oxaliplatin; CEA, carcinoembryonic antigen; MSI, microsatellite instability; MSS, microsatellite stable status; IQR, interquartile range.

**Table 2:** Univariate Cox regression analysis for PFS

	HR	95% CI	*P*-value
Age (<65 versus ≥65 years)	0.83	0.44-1.59	.5778
Gender (Male versus female)	0.92	0.49-1.73	.7862
Response to first-line treatment (SD/PD versus CR/PR)	1.96	1.02-3.79	.0440
Previous Surgery (Yes versus no)	0.46	0.24-0.90	.0223
Primary tumor location (right versus left)	2.04	1.02-4.07	.0428
Number of organ metastasis (≥2 versus 1)	2.19	1.09-4.39	.0278
CEA (<5 ng/mL versus ≥5 ng/mL)	0.41	0.21-0.80	.0088
RAS/RAF mutated (Yes versus no)	2.36	1.17-4.74	.0161
Peritoneal seeding (Yes versus no)	0.75	0.38-1.49	.4075

HR, hazard ratio; CI, confidence interval; CR, complete response; PR, partial response; SD, stable disease; PD, progressive disease; CEA, carcinoembryonic antigen.

## Abbreviations

CRC: colorectal cancer
EGFR: epidermal growth factor receptor
FOLFIRI: Fluorouracil, leucovorin, irinotecan
FOLFOX: fluorouracil, leucovorin, oxaliplatin
CAPEOX: capecitabine, oxaliplatin
OS: overall survival
PFS: progression-free survival
ECOG: Eastern Cooperative Oncology Group
CEA: carcinoembryonic antigen
CR: complete response
PR: partial response
SD: stable disease
PD: progressive disease
