# Supplementary material for: Prognostic Factors of Survival with Aflibercept and FOLFIRI (fluorouracil, leucovorin, irinotecan) as Second-line Therapy for Patients with Metastatic Colorectal Cancer
Source: J Cancer. 2021 Jan 1;12(2):460–6. doi: 10.7150/jca.49176 (PMC7739009; doi:10.7150/jca.49176)

Table S1. Best overall response to treatment

Overall response	Patient number	% (95% CI)
CR	4	7.7 (0.5-14.9)
PR	21	40.4 (27.1-53.7)
SD	19	36.5 (23.5-49.6)
PD	8	15.4 (5.6-25.2)
Overall response rate	25	48.1 (34.5-61.7)
Disease control rate	44	84.6 (74.8-94.4)

CI, confidence interval; CR, complete response; PR, partial response; SD, stable disease; PD, progressive disease.

Figure S1. Kaplan–Meier plot of PFS according to (A) baseline CEA level and (B) RAS/RAF mutation status.

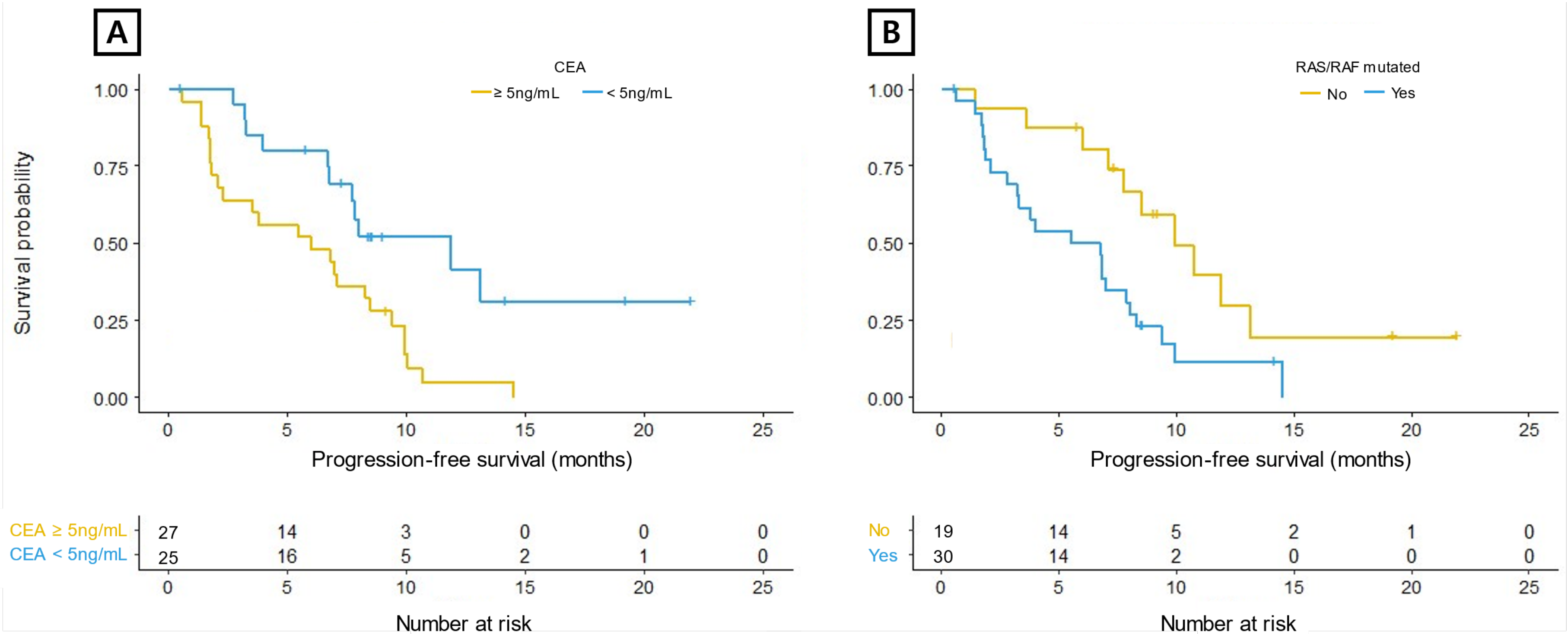

Figure S2. Kaplan–Meier plot of PFS according to (A) primary tumor location and (B) response to first-line treatment.

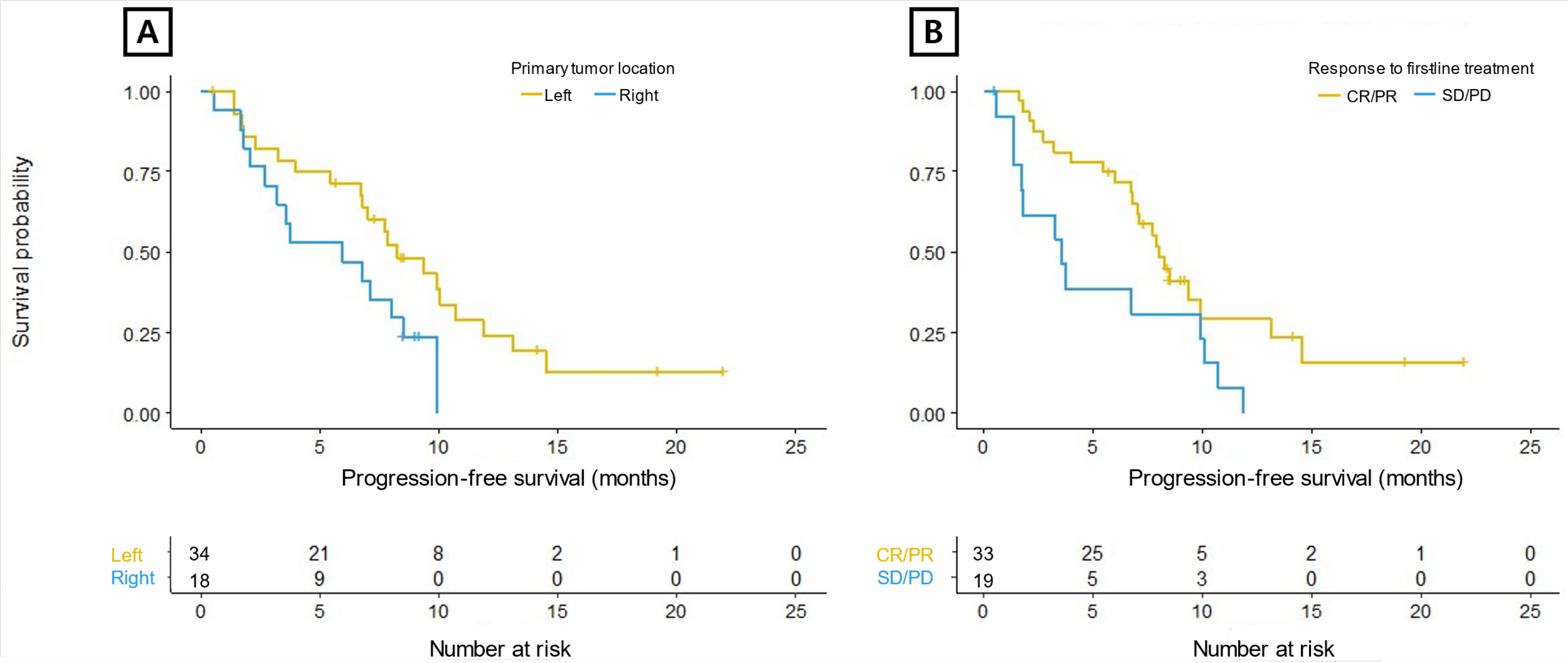

Figure S3. Kaplan–Meier plot of PFS according to (A) previous surgery and (B) number of organ metastasis.

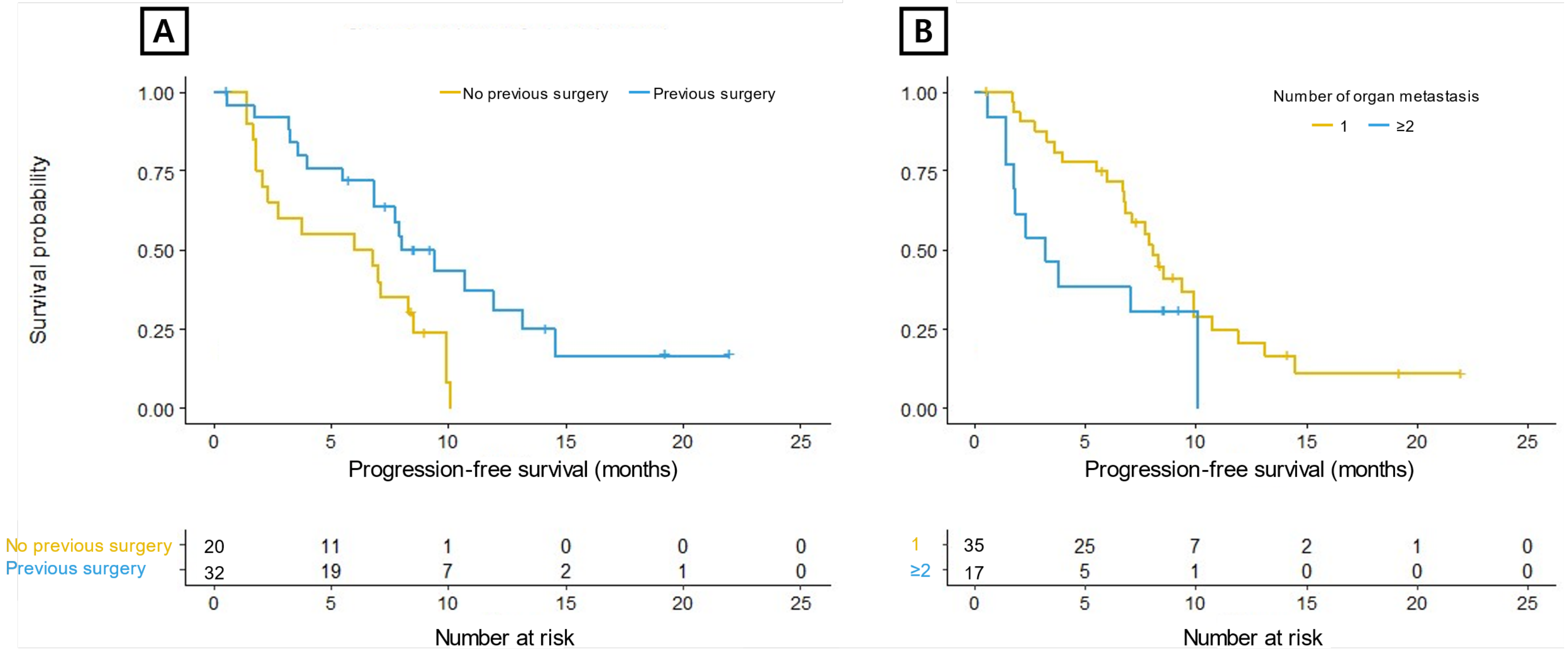

Figure S4. Multivariate survival model after variable selection

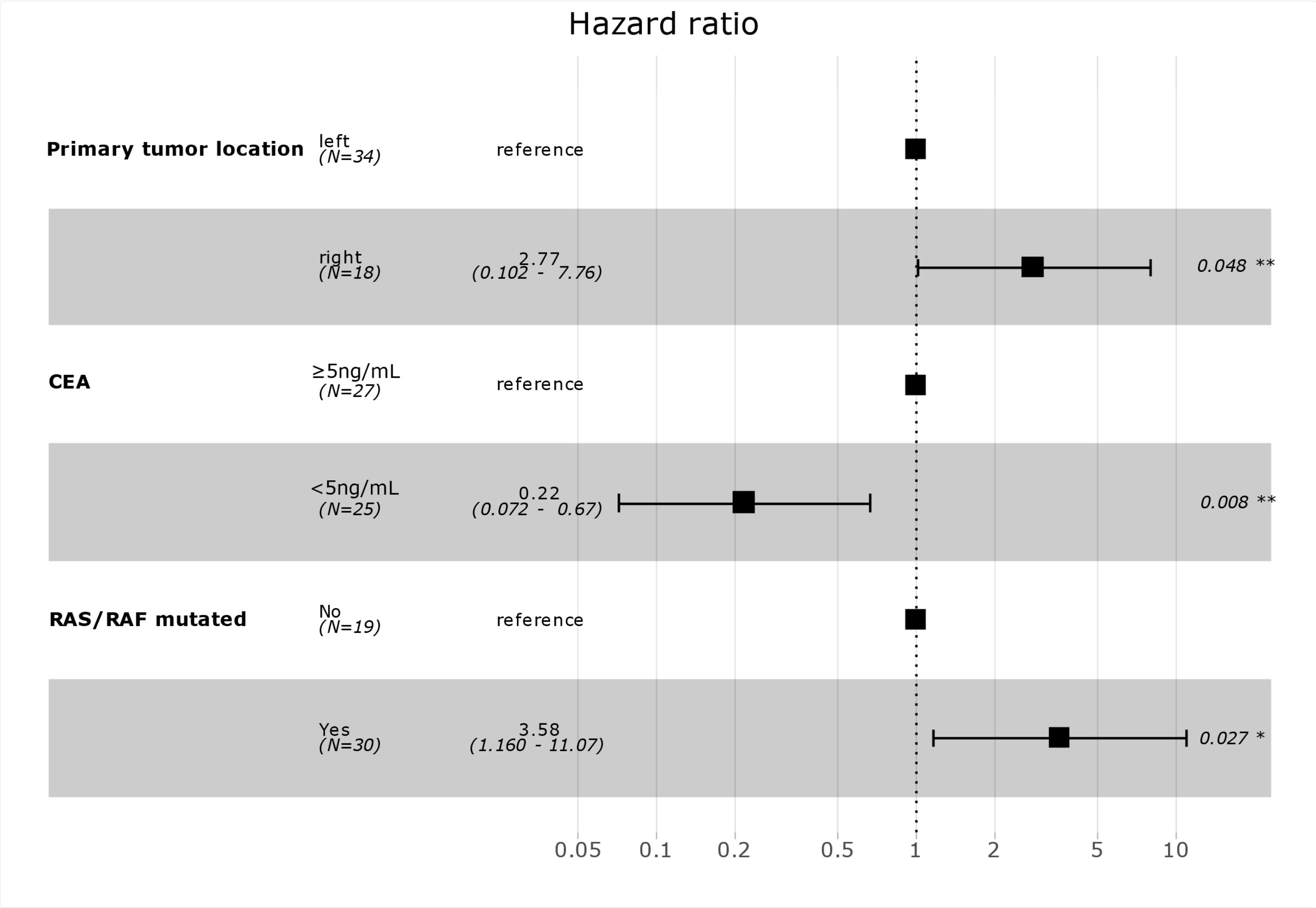

Supplement: Supplementary file 1 — Supplementary figures and tables. [file jcav12p0460s1.pdf]
